# Polaribacter ponticola sp. nov., isolated from seawater, reclassification of Polaribacter undariae as a later heterotypic synonym of Polaribacter sejongensis, and emended description of Polaribacter sejongensis Kim et al. 2013

**DOI:** 10.1099/ijsem.0.006526

**Published:** 2024-09-12

**Authors:** Ju Hye Baek, Mahrukh Butt, Dong Min Han, Jeong Min Kim, Seohui Choi, Che Ok Jeon

**Affiliations:** 1 Department of Life Science, Chung-Ang University, Seoul 06974, Republic of Korea

**Keywords:** new taxa, *Polaribacter ponticola*, *Polaribacter sejongensis*, *Polaribacter undariae*, reclassification, seawater

## Abstract

A Gram-stain-negative, yellow-pigmented, and strictly aerobic bacterium, designated as strain MSW5^T^, was isolated from seawater of the Yellow Sea in South Korea. The cells were non-motile rods exhibiting oxidase- and catalase-positive activities. Growth was observed at 15–25 °C (optimum, 25 °C) and pH 5.0–9.0 (optimum, pH 7.0–8.0) and in the presence of 1.0–5.0% (w/v) NaCl (optimum, 2.0%). Menaquinone-6 was the sole respiratory quinone, and iso-C_15 : 0_, summed feature 3 (C_16 : 1_* ω*7*c* and/or C_16 : 1_* ω*6*c*), iso-C_15 : 0_ 3-OH, and C_15 : 1_* ω*6*c* were the major cellular fatty acids. Major polar lipids included phosphatidylethanolamine, two unidentified aminolipids, and three unidentified lipids. Phylogenetic analyses based on 16S rRNA gene sequences and 92 concatenated core protein sequences revealed that strain MSW5^T^ formed a distinct lineage within the genus *Polaribacter*. The genome of strain MSW5^T^ was 3582 kb in size with a 29.1 mol% G+C content. Strain MSW5^T^ exhibited the highest similarity to *Polaribacter atrinae* WP25^T^, with a 97.9% 16S rRNA gene sequence similarity. However, the average nucleotide identity and digital DNA–DNA hybridization values were 79.4 and 23.3%, respectively, indicating that strain MSW5^T^ represents a novel species. Based on its phenotypic, chemotaxonomic, and phylogenetic characteristics, strain MSW5^T^ is proposed to represent a novel species, with the name *Polaribacter ponticola* sp. nov. The type strain is MSW5^T^ (=KACC 22340^T^=NBRC 116025^T^). In addition, whole genome sequence comparisons and phenotypic features suggested that *Polaribacter sejongensis* and *Polaribacter undariae* belong to the same species, with *P. undariae* proposed as a later heterotypic synonym of *P. sejongensis*. An emended description of *Polaribacter sejongensis* is also proposed.

## Introduction

The genus *Polaribacter*, classified within the family *Flavobacteriaceae* of the phylum *Bacteroidota*, was initially proposed by Gosink *et al*. [1] by the descriptions of *Polaribacter filamentus* (type species), *Polaribacter franzmannii*, and *Polaribacter irgensii* and the reclassification of ‘*Flectobacillus glomeratus*’ as *Polaribacter glomeratus*. Subsequent emendations of the genus *Polaribacter* were proposed by Fukui *et al*. [2], Kim *et al*. [3], and Li *et al*. [4]. At the time of writing, the genus comprises 29 species with validly published names (https://lpsn.dsmz.de/genus/polaribacter). Members of the genus *Polaribacter* have primarily been isolated from various marine-related environments, including the Antarctic Sea, seawater, algae, the intestine of the comb pen shell, tidal flats, lagoons, deep-sea polymetallic nodules, marine gastropods, and shellfish [1,15]. This genus encompasses Gram-stain-negative, aerobic, non-motile bacteria with rod-shaped morphology, typically orange or yellow in colour, and chemotaxonomically, *Polaribacter* species are characterized by menaquinone-6 (MK-6) as the major respiratory quinone, genomic DNA G+C content ranging from 28.6 to 36.4%, and phosphatidylethanolamine (PE) as the major polar lipid [1,15].

During an investigation of culturable bacteria in seawater samples collected from the Yellow Sea, a putative *Polaribacter* novel strain, designated as strain MSW5^T^, was isolated. In this study, strain MSW5^T^ was taxonomically characterized using a polyphasic approach. Additionally, we also proposed that *Polaribacter undariae* should be reclassified as a later heterotypic synonym of *Polaribacter sejongensis* based on genomic comparisons and phenotypic features.

## Strain isolation

Strain MSW5^T^ was isolated from a seawater sample collected from the intertidal zone of Baengnipo Beach in the Yellow Sea (36° 48′ 43.5″ N 126° 09′ 14.2″ E) in the Republic of Korea, as previously described [15]. Briefly, the seawater sample underwent serial dilution (10-fold dilution) in artificial seawater (ASW; 20 g NaCl, 2.9 g MgSO_4_, 4.53 g MgCl_2_‧6H_2_O, 0.64 g KCl, and 1.75 g CaCl_2_‧2H_2_O per litre). Aliquots of 0.1 ml from each serial dilution were spread on marine agar (MA; MBcell) plates and incubated aerobically at 25 °C for 7 days. Colonies grown on MA were subjected to PCR amplification of the 16S rRNA genes using universal 27F (5′-AGAGTTTGATCMTGGCTCAG-3′) and 1492R (5′-TACGGYTACCTTGTTACGACTT-3′) primers [15]. The resulting PCR products were double-digested with *Hae*III and *Hha*I, and representative amplicons with distinct fragment patterns were sequenced using the universal 340F (5′-CCTACGGGAGGCAGC AG-3′) primer [15] at Macrogen (Seoul, Republic of Korea).

The obtained 16S rRNA gene sequences were compared with those of all type strains using the Nucleotide Similarity Search program in the EzBioCloud database [16]. Strain MSW5^T^, a putative novel member of the genus *Polaribacter*, was selected for further taxonomic characterization. The isolate was routinely cultured on MA or in marine broth (MB; MBcell) for 5 days at 25 °C under aerobic conditions and preserved at −80 °C in MB supplemented with 15% (v/v) glycerol for a long-term storage. *Polaribacter atrinae* KCTC 42039^T^, *Polaribacter sejongensis* KCTC 23670^T^, *Polaribacter marinus* MSW13^T^, and *Polaribacter undariae* KCTC 42175^T^ were used as reference strains for genomic, phenotypic, and fatty acid composition comparisons.

## Phylogeny based on 16S rRNA gene sequences

The 16S rRNA gene amplicon of strain MSW5^T^, generated by primers 27F and 1492R, was further sequenced using the primers 518R (5′-ATTACCGCGGCTGCTGG-3′) and 805F (5′-GATTAGATACCCTGGTAGTC-3′) [15]. The sequences obtained from primers 340F, 518R, and 805F were assembled to get a nearly complete 16S rRNA gene sequence (1454 nucleotides). The 16S rRNA gene sequence similarities between strain MSW5^T^ and closely related type strains were calculated using EzBioCloud (http://www.ezbiocloud.net/identify) [16]. The 16S rRNA gene sequences of strain MSW5^T^ and its closely related type strains were aligned using the fast secondary-structure-aware Infernal aligner (version 1.1.4) available in the Ribosomal Database Project (RDP) [17], and phylogenetic trees with bootstrap values (1000 replications) were reconstructed in the mega11 program [18]. The Kimura two-parameter model, the nearest-neighbour-interchange heuristic search method, and complete deletion options were used for the reconstructions of neighbour-joining (NJ), maximum-likelihood (ML), and maximum-parsimony (MP) trees.

**Fig. 1.**
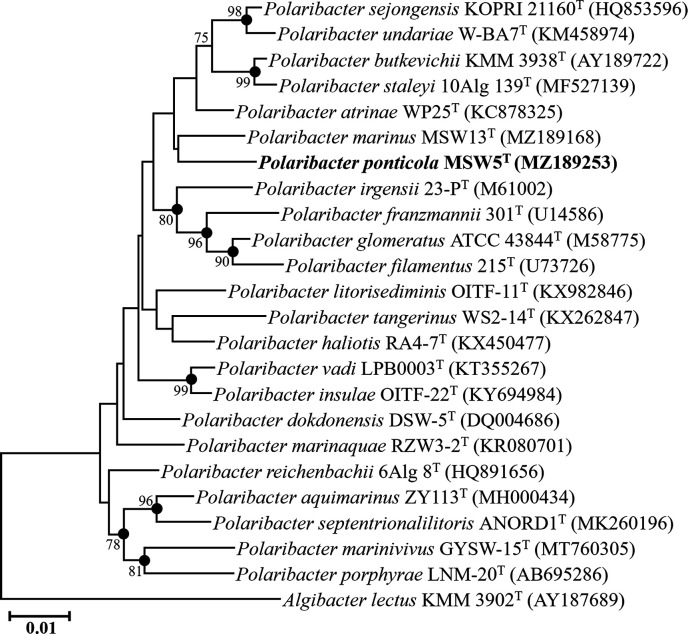
A neighbour-joining phylogenetic tree based on 16S rRNA gene sequences showing the taxonomic position of strain MSW5^T^ within the genus *Polaribacter*. Filled circles indicate the corresponding nodes (groupings) that were also recovered in the maximum-likelihood and maximum-parsimony trees. Bootstrap values (>70%) based on 1000 replicates are shown on the branch nodes. *Algibacter lectus* KMM 3902^T^ (AY187689) was used as the outgroup. Bar, 0.01 substitutions per nucleotide position.

The 16S rRNA gene sequence comparison revealed that strain MSW5^T^ exhibited the highest 16S rRNA gene sequence similarity to *P. atrinae* WP25^T^ (97.9%), followed by *P. sejongensis* KOPRI 21160^T^ (97.9%), *P. marinus* MSW13^T^ (97.8%), and *P. undariae* W-BA7^T^ (97.4%). These values fell below the species differentiation threshold of 98.7% [19], suggesting that strain MSW5^T^ likely represents a novel species. Phylogenetic analysis based on the 16S rRNA gene sequences using the NJ algorithm showed that strain MSW5^T^ formed a distinct phyletic lineage with *P. marinus* MSW13^T^ within the genus *Polaribacter* (Fig. 1). Additionally, phylogenetic trees generated using the ML and MP algorithms further confirmed the distinctiveness of strain MSW5^T^ within the genus *Polaribacter* (Fig. S1). These combined comparative and phylogenetic analyses based on 16S rRNA gene sequences support the classification of strain MSW5^T^ as a novel member of the genus *Polaribacter*.

## Whole genome sequencing and genome-based phylogeny

The genomic DNA of strain MSW5^T^ was extracted from cells cultured in MB using a Wizard Genomic DNA purification kit (Promega) and sequenced using an Oxford Nanopore MinION sequencer (ONT). The resulting sequencing reads were *de novo*-assembled using Flye (version 2.9.1) [20], and the quality of the assembled genome was assessed based on completeness and contamination rates using the CheckM program (version 1.0.4) [21]. For genome-based phylogenetic analysis, 92 single-copy core genes were extracted from the genomes of strain MSW5^T^ and its closely related type strains using the up-to-date bacterial core genes (UBCG) pipeline (https://www.ezbiocloud.net/tools/ubcg) [22]. The amino acid sequences were concatenated, aligned, and used to reconstruct a phylogenomic ML tree with bootstrap values (1000 replicates) within the UBCG pipeline. The final phylogenomic ML tree was visualized using the mega11 program. Average nucleotide identity (ANI) and digital DNA–DNA hybridization (dDDH) values among the genomes of strain MSW5^T^ and closely related type strains were calculated using the Orthologous Average Nucleotide Identity Tool software available in the EzBioCloud server (https://www.ezbiocloud.net/sw/oat) [23] and the server-based Genome-to-Genome Distance Calculator version 2.1 (https://ggdc.dsmz.de/distcalc2.php) [24], respectively.

The *de novo* assembly of the genome sequencing data derived from strain MSW5^T^ yielded a draft genome with a size of approximately 3582 kb, comprising five contigs with an N50 value of 2448 kb (Table 1). The 16S rRNA gene sequence identified in the genome of strain MSW5^T^ was consistent with that obtained by PCR-based sequencing. The completeness and contamination rate of the genomes of strain MSW5^T^ were 90.5 and 4.6%, respectively, meeting the criteria representing generally high-quality genomes (completeness ≥90% and contamination ≤10%) [21].

**Table 1.** General genomic features and genome relatedness of strain MSW5^T^ and the type strains of closely related *Polaribacter* species Strains: 1, MSW5^T^ (JAOSLC000000000); 2, *P. atrinae* KACC 17473^T^ (LVWE00000000); 3, *P. marinus* MSW13^T^ (JAKQYM000000000); 4, *P. sejongensis* KCTC 23670^T^ (CP019336); 5, *P. undariae* KCTC 42175^T^ (CP103460).

Feature*	1	2	3	4	5
Genome size (kb)	3582	3943	3393	4526	4418
G+C content (mol%)	29.1	31.2	29.9	30.5	30.7
N50 (kb)	2448	219	297	4526	4418
No. of contigs	5	113	50	1	1
No. of total genes	3454	3416	3070	3655	3657
No. of coding sequences	3401	3366	3028	3611	3587
No. of total RNA genes	53	50	42	44	70
No. of tRNA genes	40	42	35	40	51
No. of noncoding RNA genes	4	4	4	4	4
No. of rRNA genes (5S, 16S, 23S)	3, 3, 3	2, 1, 1	1, 1, 1	–	5, 5, 5
No. of confirmed CRISPRs†	1	4	1	–	–
No. of total CAZyme† genes:	107	135	83	189	187
Glycoside hydrolase	50	73	25	107	105
Glycosyltransferase	27	40	38	46	38
Polysaccharide lyase	5	3	5	13	17
Carbohydrate esterase	14	12	11	14	16
Auxiliary activities	3	1	1	0	0
Carbohydrate-binding modules	8	6	3	9	11

*The information was obtained from GenBank annotated by the NCBI prokaryotic genome annotation pipeline (www.ncbi.nlm.nih.gov/genome/annotation_prok/).

†CRISPR, clustered regularly interspaced short palindromic repeat; CAZyme, Carbohydrate-Active enZYmes.

**Fig. 2.**
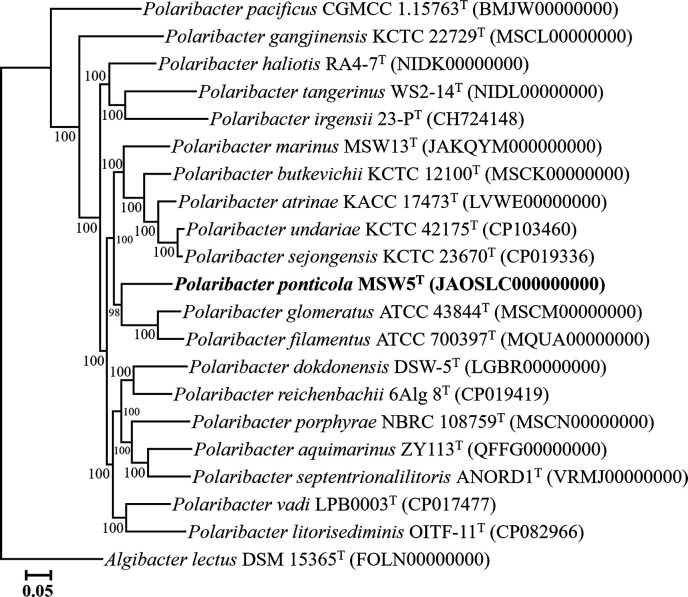
A maximum-likelihood phylogenomic tree showing the phylogenetic relationships between strain MSW5^T^ and their closely related taxa, based on the concatenated amino acid sequences of 92 house-keeping core genes. Bootstrap values (>70%) based on 1000 replicates are shown on the branch nodes. *Algibacter lectus* DSM 15365^T^ (FOLN00000000) was used as the outgroup. Bar, 0.05 changes per amino acid position.

The phylogenetic tree based on the concatenated 92 bacterial core protein sequences showed that strain MSW5^T^ formed a distinct phylogenetic lineage within the genus *Polaribacter* (Fig. 2), supporting that strain MSW5^T^ can be assigned to a novel species of the genus *Polaribacter*. The ANI and dDDH values between strain MSW5^T^ and the closely related type strains were less than 80.1 and 23.8%, respectively (Table S1, available in the online Supplementary Material), which were clearly lower than the thresholds (ANI, ~95–96%; dDDH, 70%) for prokaryotic species delineation [19]. The results from the phylogenomic analysis and genome relatedness assessments strongly support the conclusion that strain MSW5^T^ represents a novel species of the genus *Polaribacter*.

## Genomic features

The genomes of strain MSW5^T^ (JAOSLC000000000), *P. atrinae* KACC 17473^T^ (LVWE00000000), *P. marinus* MSW13^T^ (JAKQYM000000000), *P. sejongensis* KCTC 23670^T^ (CP019336), and *P. undariae* KCTC 42175^T^ (CP103460) were analysed using the NCBI Prokaryotic Genome Annotation Pipeline [25]. To identify genes linked with Carbohydrate Active enZymes (CAZymes) within these genomes, the automated carbohydrate-active enzyme annotation web server, dbCAN 3.0 (https://bcb.unl.edu/dbCAN2/blast.php) [26], was employed.

Strain MSW5^T^ was found to possess a total of 3454 genes, comprising 3401 coding sequences, 53 total RNA genes, 40 tRNA genes, four noncoding RNA genes, and three rRNA operons containing three 5S, three 16S, and three 23S rRNA genes. These characteristics closely resembled those observed in other closely related *Polaribacter* species (Table 1). The DNA G+C content of strain MSW5^T^, derived from the draft whole genome sequence, was calculated to be 29.1 mol%, consistent with the G+C contents observed in closely related *Polaribacter* strains. A summary of the general genomic features of strain MSW5^T^ is provided in Table 1, along with a comparison with those of closely related *Polaribacter* type strains.

In marine environments, the abundant production of polysaccharides by diverse algae highlights the critical role of marine bacteria in degrading these compounds for survival. Previous studies have emphasized the polysaccharide degradation abilities of numerous *Polaribacter* species, particularly those isolated from marine algae [27,28]. Therefore, we conducted a comprehensive genome-wide analysis of genes encoding CAZymes in strain MSW5^T^ and four closely related *Polaribacter* type strains (Table 1). Our analysis, based on the CAZy database, identified a total of 107 putative CAZyme-encoding genes in the genome of strain MSW5^T^. Notably, the reference *Polaribacter* strains also exhibited a substantial presence of CAZyme-encoding genes, indicating the versatile capabilities of *Polaribacter* species in decomposing diverse algal polysaccharides. Comparative analysis revealed that strain MSW5^T^ possessed fewer total CAZyme genes compared to reference strains such as *P. atrinae* (135), *P. sejongensis* (189), and *P. undariae* (187). However, it harboured more CAZyme genes than *P. marinus* (83), which was also isolated from seawater. This suggests that strain MSW5^T^, along with *P. marinus*, may exhibit relatively lower capabilities in degrading algal polysaccharides compared to other reference strains.

## Morphology and phenotypic properties

Growth of strain MSW5^T^ was assessed on several bacteriological agar media, including MA, R2A agar (MBcell), Luria–Bertani agar prepared in the laboratory, tryptic soy agar (MBcell), and nutrient agar (MBcell) containing 2.0% (w/v) NaCl at 25 °C for 5 days. Colony morphology was observed on MA after 5 days of incubation at 25 °C. The growth temperature and pH range of strain MSW5^T^ were investigated at different temperatures (ranging from 5 to 40 °C at 5 °C intervals) and pH values (ranging from pH 4.0 to 11.0 at 1.0 pH unit intervals) on MA and in MB, respectively. MB media with desired pH levels were prepared using sodium citrate (pH 4.0–5.0), Na_2_HPO_4_/NaH_2_PO_4_ (pH 6.0–8.0), and sodium carbonate-bicarbonate (pH 9.0–11.0) buffers [29], followed by pH readjustment post-sterilization (121 °C for 15 min) if necessary. NaCl tolerance was determined at 25 °C for 5 days in MB with varying NaCl concentrations (ranging from 0–10% at 0.5% intervals, w/v) prepared according to the MB composition. Anaerobic growth of strain MSW5^T^ was assessed on MA at 25 °C for 21 days under anaerobic conditions created by the GasPak Plus system (BBL). Cell morphology and flagellum motility were examined by a JEM-1010 transmission electron microscope (jeol) and a phase-contrast microscope (Carl Zeiss) with cells grown on MA at 25 °C for 5 days. The gliding motility of strain MSW5^T^ was tested using MA containing 0.3% (w/v) agar, following a previously described method [30]. Gram staining was performed using a Gram stain kit (bioMérieux), following the manufacturer’s instructions. Catalase activity was determined by observing bubble production in 3% (v/v) hydrogen peroxide, and oxidase activity was assessed colorimetrically using 1% (w/v) tetramethyl-*p*-phenylenediamine (Merck) [31]. Flexirubin-type pigment production was evaluated as described by Bernardet *et al*. [30]. Hydrolysis of casein (1% skimmed milk, w/v), starch (1%, w/v), aesculin (0.1%, w/v), l-tyrosine (0.5%, w/v), Tween 20 (1%, w/v), and Tween 80 (1%, w/v) was assayed on MA, as described previously [32]. Further enzymatic activities and biochemical features were assessed using the API 20NE and API ZYM kits (bioMérieux), following the manufacturer’s instructions, except that inocula were prepared by resuspending cells in ASW.

Strain MSW5^T^ exhibited robust growth on MA; however, it failed to grow on R2A agar, nutrient agar, LB agar, and tryptic soy agar containing 2.0% NaCl. The cells of strain MSW5^T^ were characterized as Gram-stain-negative, non-motile rods, measuring approximately 0.7–0.8 µm wide and 1.6–2.6 µm long (Fig. S2). Anaerobic growth was not observed following 21 days of incubation on MA at 25 °C, and flexirubin-type pigments were absent (KOH-negative). Several phenotypic traits of strain MSW5^T^, such as catalase and oxidase positivity, flexirubin-type pigment production, absence of indole production and nitrate reduction, and casein hydrolysis, were consistent with those observed in closely related reference type strains. However, certain phenotypic characteristics, including growth range and inability to hydrolyse aesculin, Tween 20, and l-tyrosine, differentiated strain MSW5^T^ from closely related *Polaribacter* type strains (Table 2).

**Table 2.** Differential phenotypic characteristics of strain MSW5^T^ and the type strains of closely related species in the genus *Polaribacter* Strains: 1, MSW5^T^ (this study); 2, *P. atrinae* KCTC 42039^T^; 3, *P. marinus* MSW13^T^; 4, *P. sejongensis* KCTC 23670^T^; 5, *P. undariae* KCTC 42175^T^. All strains were positive for the following characteristics: activity* of oxidase, catalase, alkaline phosphatase, esterase (C4), esterase lipase (C8), leucine arylamidase, valine arylamidase, acid phosphatase, cystine arylamidase, and naphthol-AS-BI-phosphohydrolase. All strains are negative for the following characteristics: motility, indole production, nitrate reduction, flexirubin-type pigment production, casein hydrolysis*, activity* of lipase (C14), *α*-chymotrypsin, *α*-glucosidase, *β*-glucuronidase, *N*-acetyl-*β*-glucosaminidase, *α*-mannosidase, *α*-fucosidase, urease, and arginine dihydrolase, and assimilation* of d-glucose, l-arabinose, d-mannose, d-mannitol, potassium gluconate, capric acid, adipic acid, malic acid, *N*-acetyl-glucosamine, maltose, trisodium citrate, and phenylacetic acid. Symbols: +, positive; –, negative; w, weakly positive.

Characteristic	1	2	3	4	5
Isolation source	Seawater	Intestine of comb pen shell	Seawater	Antarctic soil	Brown alga reservoir
Colony colour on MA*	Yellow	Yellow	Yellow	Light yellow	Pale yellow
Anaerobic growth	–	–	+	–	–
Growth range (optimum) of:					
Temperature (°C)	15–25 (25)	4–30 (20)	4–30 (25)	4–37 (25)	4–30 (25)
NaCl (%)	1–5 (2)	1–6 (2)	1–5 (2)	0.5–5 (3)	0.5–6 (2)
pH	5–9 (7–8)	5–9 (7)	5–9 (6-7)	7–8.5(7.5)	5.5–9 (7–8)
Glucose fermentation*	–	w	–	+	+
Acetoin production*	–	+	–	+	w
Hydrolysis* of:					
Starch	–	+	–	+	+
Aesculin, l-tyrosine, Tween 20	–	+	+	+	+
Gelatin	w	–	+	+	+
Tween 80	+	+	–	+	+
Enzyme activity* of:					
*β*-Galactosidase	+	+	–	+	+
Trypsin	–	w	+	w	–
*α*-Galactosidase	w	+	–	+	+
*β*-Glucosidase	–	–	–	+	–
Polar lipids†	PE, 2ALs, 3Ls	PE, 2ALs, PL, 4Ls	PE, 3ALs, 4Ls	PE, 2ALs, L	PE, AL, GL, 3Ls

*All data were obtained in this study.Data, excluding the results obtained in this study, were sourced from the following references: Hyun *et al*. [6] for *P. atrinae*, Kristyanto *et al*. [15] for *P. marinus*, Kim *et al*. [3] for *P. sejongensis*, and Park *et al*. [12] for *P. undariae*.

*All data were obtained in this study.

†PE, phosphatidylethanolamine; AL, unidentified aminolipid; GL; unidentified glycolipid; L, unidentified lipid.

## Chemotaxonomy

Isoprenoid quinones of strain MSW5^T^ were extracted from cells cultivated in MB at 25 °C until reaching the exponential growth phase, following the procedure outlined by Minnikin *et al*. [33] and analysed using an HPLC system (LC-20A, Shimadzu) equipped with a reversed-phase Kromasil column (250×4.6 mm; Akzo Nobel Center) and an SPD-M20A diode array detector (Shimadzu). Methanol–isopropanol (2 : 1, v/v) was used as the eluent, and the flow rate was 1 ml min^−1^. For cellular fatty acid analysis, strain MSW5^T^ and reference strains were aerobically cultivated in MB at 25 °C, and their microbial cells were harvested during the exponential growth stage (OD_600_=0.7–0.8). Cellular fatty acids were saponified, methylated, and extracted according to the standard procedure described by midi (Sherlock Microbial Identification System, version 6.2B). Fatty acid methyl esters were analysed using a 6890-gas chromatograph (Hewlett Packard) and identified using the RTSBA6 database of the Microbial Identification System Sherlock version 6.0B [34]. Polar lipids of strain MSW5^T^ were extracted from cells harvested during the exponential growth phase and analysed by two-dimensional TLC, following the procedure by Minnikin *et al.* [35]. Various reagents were employed to identify different polar lipids: 10% ethanolic molybdophosphoric acid (for total polar lipids), ninhydrin (for aminolipids), Dittmer–Lester reagent (for phospholipids), and *α*-naphthol/sulphuric acid (for glycolipids). The presence of PE in strain MSW5^T^ was confirmed using authentic PE purchased from Sigma-Aldrich.

MK-6 was identified as the sole respiratory quinone in strain MSW5^T^, consistent with the characteristics of members of the genus *Polaribacter* [1,15]. The major cellular fatty acids (>10%) detected in strain MSW5^T^ were iso-C_15 : 0_ (19.7%), summed feature 3 (C_16 : 1_* ω*7*c* and/or C_16 : 1_* ω*6*c*; 12.5%), iso-C_15 : 0_ 3-OH (12.4%), and C_15 : 1_* ω*6*c* (12.2%) (Table S2). Although the overall fatty acid profile of strain MSW5^T^ was generally similar to those of closely related *Polaribacter* type strains, some differences in some components were noted. For example, anteiso-C_15 : 0_ was identified as a minor fatty acid in strain MSW5^T^, whereas it was predominant in closely related *Polaribacter* species, *P. atrinae* KCTC 42039^T^ and *P. marinus* MSW13^T^. Conversely, summed feature 3 was abundant in strain MSW5^T^ but minor in *P. atrinae* KCTC 42039^T^ and *P. marinus* MSW13^T^, thereby distinguishing strain MSW5^T^ from closely related *Polaribacter* species. In strain MSW5^T^, PE, along with two unidentified aminolipids and three unidentified lipids, were identified as the major polar lipids (Fig. S3). The identification of PE as a major polar lipid in strain MSW5^T^ was consistent with observations in other *Polaribacter* species (Table 2).

## Taxonomic conclusion

In conclusion, the phylogenetic, genomic relatedness, phenotypic, physiological, biochemical, and chemotaxonomic features strongly support that strain MSW5^T^ represents a novel species of the genus *Polaribacter*, for which the name *Polaribacter ponticola* sp. nov. is proposed.

## Reclassification of *Polaribacter undariae* as a later heterotypic synonym of *Polaribacter sejongensis*

The 16S rRNA gene sequence-based NJ, ML, and MP trees and genome-based phylogenomic tree clearly showed that two type strains proposed differently as *P. sejongensis* and *P. undariae* [3,12] formed a tight phyletic lineage within the genus *Polaribacter* (Figs1  2, and S1). The 16S rRNA gene sequence similarity between their type strains was 99.3%, and the ANI and dDDH values between the genomes of *P. undariae* KCTC 42175^T^ (CP114178) and *P. sejongensis* KCTC 23670^T^ (CP019336) were 97.5 and 76.8%, respectively (Table S1), which were clearly higher than the thresholds for prokaryotic species delineation [19]. In addition, the comparison of phenotypic, chemotaxonomic, and genomic features showed that the type strains of *P. sejongensis* and *P. undariae* had similar phenotypic characteristics, including hydrolysis of carbon compounds, enzyme activities, major polar lipids, G+C contents, and major fatty acids (Tables1  2 and S2). These results suggest that *P. sejongensis* and *P. undariae* belong to the same species of the genus *Polaribacter*, and thus in this study, it is proposed that *Polaribacter undariae* Park *et al*. 2015 is reclassified as a later heterotypic synonym of *Polaribacter sejongensis* Kim *et al*. 2013.

## Description of *Polaribacter ponticola* sp. nov.

*Polaribacter ponticola* (pon.ti′co.la. L. masc. n. *pontus*, the sea; L. masc./fem. n. suff. -*cola*, a dweller, inhabitant; N.L. masc. n. *ponticola*, a dweller of sea).

Colonies are yellow, smooth, and circular with entire edges and slightly convex with approximately 0.5–1.0 mm in diameter after 5 days of incubation at 25 °C on MA. Cells are Gram-stain-negative, strictly aerobic, and non-motile rods (approximately 0.7–0.8 µm wide and 1.6–2.6 µm long). Gliding motility is negative. Growth occurs at 15–25 °C (optimum, 25 °C) and pH 5.0–9.0 (optimum, pH 7.0–8.0) and in the presence of 1–5% (w/v) NaCl (optimum, 2.0% NaCl). Flexirubin-type pigments are not produced. Oxidase- and catalase-positive. Tween 80 and gelatin (weakly) are hydrolysed, but casein, starch, aesculin, Tween 20, and l-tyrosine are not. Nitrate is not reduced. Indole production and d-glucose fermentation are negative. Alkaline phosphatase, esterase (C4), esterase lipase (C8), leucine arylamidase, valine arylamidase, cystine arylamidase, acid phosphatase, naphthol-AS-BI-phosphohydrolase, *α*-galactosidase (weakly), and *β*-galactosidase activities are positive, but arginine dihydrolase, urease, lipase (C14), trypsin, *α*-chymotrypsin, *α*-glucosidase, *β*-glucosidase, *β*-glucuronidase, *N*-acetyl-*β*-glucosaminidase, *α*-mannosidase, and *α*-fucosidase activities are negative. Assimilation of d-glucose, l-arabinose, maltose, d-mannose, d-mannitol, *N*-acetyl-glucosamine, potassium gluconate, adipic acid, capric acid, malic acid, trisodium citrate, and phenylacetic acid is negative. The major fatty acids (>10% of the total fatty acids) are iso-C_15 : 0_, summed feature 3 (C_16 : 1_* ω*7*c* and/or C_16 : 1_* ω*6*c*), iso-C_15 : 0_ 3-OH, and C_15 : 1_* ω*6*c*. Menaquinone-6 is the sole respiratory quinone. Phosphatidylethanolamine, two unidentified aminolipids, and three unidentified lipids are identified as polar lipids.

The type strain is MSW5^T^ (=KACC 22340^T^=NBRC 116025^T^), isolated from seawater of the Yellow Sea, Republic of Korea. The genome size and DNA G+C content of the type strain are 3582 kb and 29.1 mol%, respectively. The GenBank accession numbers of the 16S rRNA gene and genome sequences of strain MSW5^T^ are MZ189253 and JAOSLC000000000, respectively.

## Emended description of *Polaribacter sejongensis* Kim *et al*. 2013

Heterotypic synonym: *Polaribacter undariae* Kim *et al*. 2015.

The species description is as given for *Polaribacter sejongensis* [3] with the following amendments. The G+C content of the genomic DNA is 30.5–30.7 mol%. Major fatty acids are iso-C_15 : 0_ and iso-C_15 : 1_ G. The identification of C_15 : 1_* ω*6*c*, iso-C_15 : 0_ 3-OH, and summed feature 3 (C_16 : 1_* ω*7*c* and/or C_16 : 1_* ω*6*c*) as major fatty acids varies depending on the strain. The type strain is KOPRI 21160^T^ (=KCTC 23670^T^=JCM 18092^T^). The GenBank accession numbers for the 16S rRNA gene and genome sequences of the type strain are HQ853596 and CP019336, respectively.

## supplementary material

10.1099/ijsem.0.006526 Uncited Supplementary Material 1.

## Abbreviations

ANI: average nucleotide identity
ASW: artificial seawater
dDDH: digital DNA–DNA hybridization
MA: marine agar
MB: marine broth
MK-6: menaquinone-6
ML: maximum-likelihood
MP: maximum-parsimony
NJ: neighbour-joining
PE: phosphatidylethanolamine
