# Supplementary material for: Polaribacter ponticola sp. nov., isolated from seawater, reclassification of Polaribacter undariae as a later heterotypic synonym of Polaribacter sejongensis, and emended description of Polaribacter sejongensis Kim et al. 2013
Source: Int J Syst Evol Microbiol. 2024 Sep 12;74(9):006526. doi: 10.1099/ijsem.0.006526 (PMC11392042; doi:10.1099/ijsem.0.006526)
Supplement: Uncited Supplementary Material 1. [file ijsem-74-06526-s001.pdf]

Supplementary information

Fig. S1 Maximum likelihood (A) and maximum parsimony (B) trees showing the phylogenetic relationships between strain MSW5^T and closely related taxa, based on 16S rRNA gene sequences. Numbers on the branch nodes indicate the percentages of 1000 bootstrap replicates; only values >70% are shown. *Algibacter lectus* KMM 3902^T (AY187689) was used as the outgroup. Scale bars in panels A and B represent changes per nucleotide position and over the whole sequences, respectively.

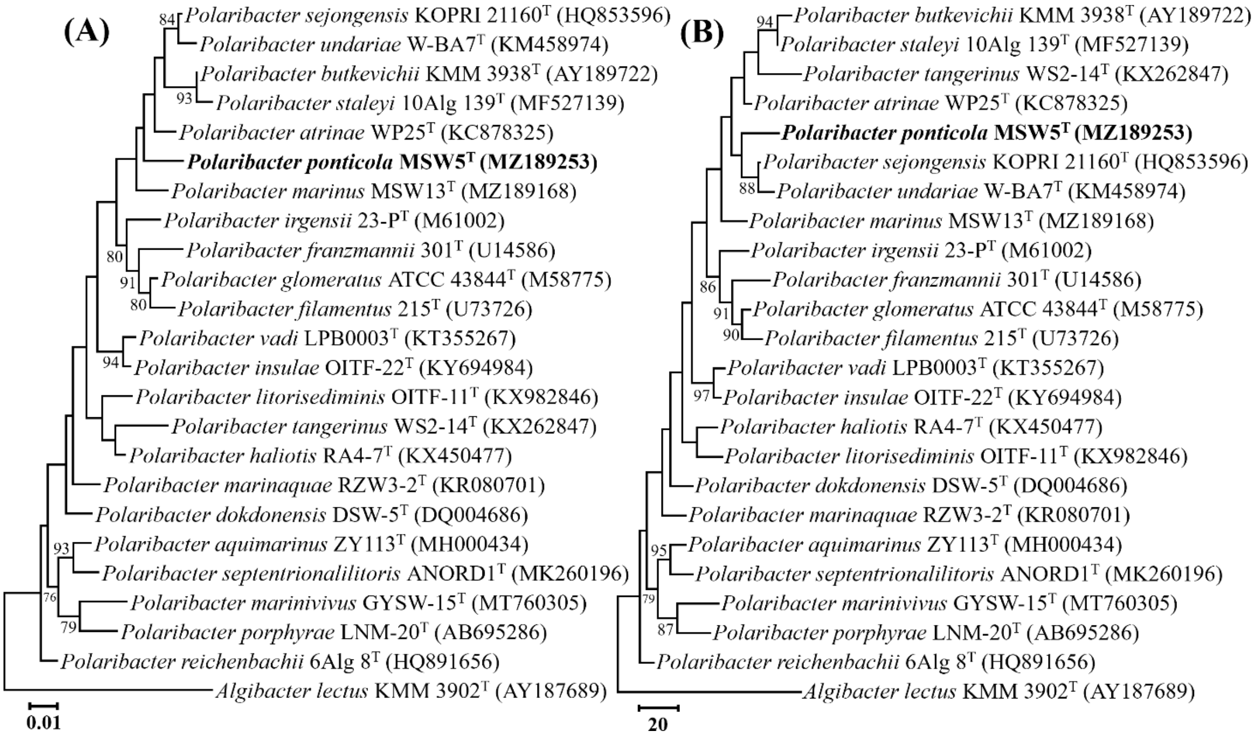

Fig. S2. Transmission electron micrographs of negatively stained cells using 2% uranyl acetate showing the general cellular morphology of strain MSW5^T. Cells grown on marine agar for 5 days at 25°C were used for the analysis. Bars, 500 nm.

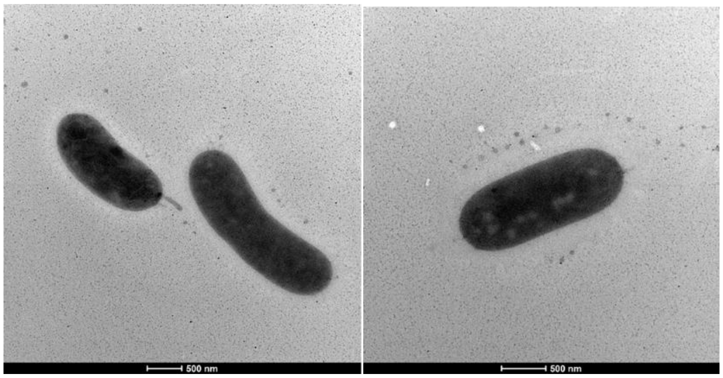

Fig. S3. Two-dimensional thin-layer chromatograms (TLC) showing the polar lipid profiles of strain MSW5^T. Solvent systems: (I) chloroform-methanol-water (65:25:4, v/v/v); (II) chloroform-acetic acid methanol-water (80:15:12:4, v/v/v/v). The TLC plates were sprayed with 10% ethanolic molybdatophosphoric acid (A), ninhydrin (B), Dittmer-Lester (C), and α-naphthol (D) reagents for the detection of total polar lipids, aminolipids, phospholipids, and glycolipids, respectively. Abbreviations: PE, phosphatidylethanolamine; AL1–2, unidentified aminolipids; L1–3, unidentified lipids.

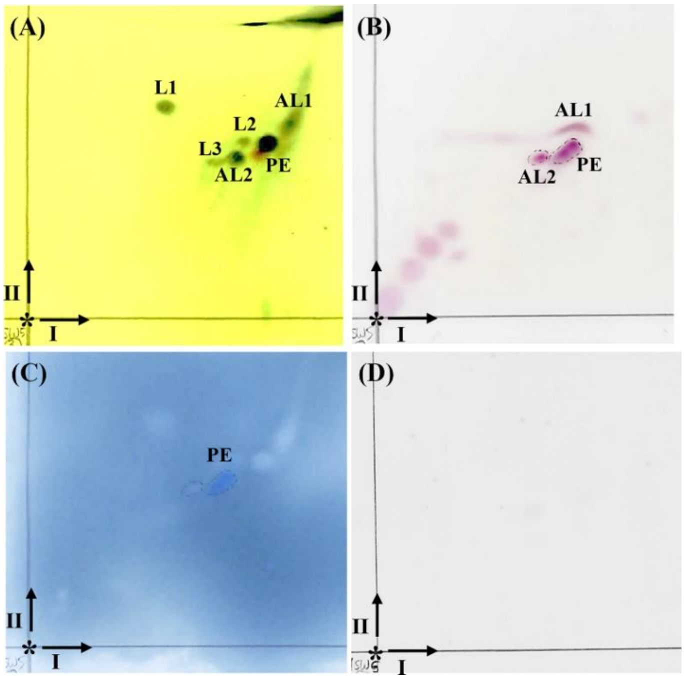

Table S1. Genome relatedness among strain MSW5^T and the type strains of closely related *Polaribacter* species

Taxa: 1, MSW5^T (JAOSLC000000000); 2, *P. atrinae* KACC 17473^T (LVWE00000000); 3, *P. marinus* MSW13^T (JAKQYM000000000); 4, *P. sejongensis* KCTC 23670^T (CP019336); 5, *P. undariae* KCTC 42175^T (CP103460).

		dDDH† value (%)				
		**1**	**2**	**3**	**4**	**5**
ANI† value (%)	**1**	–	23.3	23.3	23.4	23.8
	**2**	79.4	–	23.8	34.7	34.7
	**3**	80.2	80.5	–	24.0	23.9
	**4**	79.3	88.0	80.3	–	76.8
	**5**	79.5	88.1	80.4	97.5	–

†ANI, average nucleotide identity; dDDH, digital DNA-DNA hybridization.

Table S2. Cellular fatty acid compositions (%) of strain MSW5^T and the type strains of closely related *Polaribacter* species

Taxa: 1, MSW5^T; 2, *P. atrinae* KCTC 42039^T; 3, *P. marinus* MSW13^T; 4, *P. sejongensis* KCTC 23670^T; 5, *P. undariae* KCTC 42175^T. All data were obtained from this study. Data are expressed as percentages of the total fatty acids, and fatty acids amounting less than 1.0% in all strains are not shown. Major components (>10.0%) are highlighted in bold; tr, trace amount (<1.0%); –, not detected.

**Fatty acid**	**1**	**2**	**3**	**4**	**5**
Saturated:					
$C_{13:0}$	tr	tr	tr	1.1	tr
$C_{14:0}$	tr	tr	tr	1.2	tr
Unsaturated:					
$C_{15:1}$ $\omega 6c$	**12.2**	**16.2**	**14.2**	9.2	1.1
$C_{17:1}$ $\omega 6c$	tr	2.2	2.0	tr	tr
anteiso-$C_{17:1}$ $\omega 9c$	–	–	–	–	1.9
Branched:					
iso-$C_{13:0}$	3.2	4.3	5.2	6.2	tr
iso-$C_{14:0}$	6.5	3.5	4.1	2.7	1.1
iso-$C_{15:0}$	**19.7**	**15.7**	**14.7**	**11.4**	**19.0**
iso-$C_{16:0}$	tr	tr	tr	tr	1.8
iso-$C_{15:1}$ G	9.5	4.6	5.5	**14.1**	**15.1**
iso-$C_{16:1}$ H	2.5	2.1	1.7	tr	4.0
anteiso-$C_{15:0}$	1.9	**17.8**	**18.7**	2.3	6.8
anteiso-$C_{15:1}$ A	–	tr	1.1	tr	4.6
Hydroxy:					
$C_{15:0}$ 2-OH	tr	1.7	1.9	tr	2.5
$C_{15:0}$ 3-OH	4.8	5.9	6.3	3.3	–
$C_{16:0}$ 3-OH	tr	tr	tr	3.2	tr
$C_{17:0}$ 2-OH	tr	tr	tr	–	2.6
iso-$C_{15:0}$ 3-OH	**12.4**	**10.3**	**11.9**	**11.9**	7.2
iso-$C_{16:0}$ 3-OH	5.5	2.9	2.9	2.8	7.4
iso-$C_{17:0}$ 3-OH	1.8	1.7	1.5	4.4	6.6
Summed features*:					
3	**12.5**	2.6	2.2	**17.7**	7.1
9	tr	tr	tr	tr	6.9

*Summed features are fatty acids that cannot be resolved reliably from another fatty acid using the chromatographic conditions chosen. The MIDI system groups these fatty acids together as one feature with a single percentage of the total. Summed features 3 and 9 comprise $C_{16:1}$ $\omega 7c$ and/or $C_{16:1}$ $\omega 6c$ and $C_{16:0}$ 10-methyl and/or iso-$C_{17:1}$ $\omega 9c$, respectively.
